# Patient Life Engagement and Metabolic Profile Improve After Switching from First-/Second-Generation Antipsychotics to Brexpiprazole: A Real-World Study in Patients with Schizophrenia

**DOI:** 10.3390/jpm15110502

**Published:** 2025-10-22

**Authors:** Marco Di Nicola, Maria Pepe, Miriam Milintenda, Marco Massetti, Lorenzo Moccia, Isabella Panaccione, Gabriele Sani

**Affiliations:** 1 Department of Neuroscience, Section of Psychiatry, Università Cattolica del Sacro Cuore, 00168 Rome, Italy; 2 Department of Psychiatry, Fondazione Policlinico Universitario ‘Agostino Gemelli’ IRCCS, 00168 Rome, Italy; 3 Mental Health Department, ASL Roma 1, 00193 Rome, Italy

**Keywords:** psychosis, brexpiprazole, pharmacological switch, life engagement, personalized medicine

## Abstract

**Background:** Schizophrenia is a chronic disorder requiring long-term pharmacological treatment. Many patients experience inadequate response and adverse effects, often leading to poor adherence and need for antipsychotic switch or polypharmacotherapy. In this context, brexpiprazole, an atypical antipsychotic with favorable tolerability profile, may offer clinical benefits following previous treatment failure or intolerance. However, real-world evidence after treatment switch remains limited. **Methods**: This retrospective, observational study included 50 outpatients with schizophrenia switched to brexpiprazole (2–4 mg/day) via cross-titration and evaluated over 12 weeks. Primary outcomes were changes in *Patient Life Engagement*, assessed through a 14-item subset of the Positive and Negative Syndrome Scale (PANSS), along with response/remission rates. Secondary outcomes included changes in subjective well-being, quality of life, sexual functioning (based on Subjective Well-being under Neuroleptics—Short Form [SWN-S], WHO-5 Well-Being Index [WHO-5], and Arizona Sexual Experience Scale [ASEX] scores, respectively), metabolic parameters, and prolactin levels. **Results**: Life engagement improved significantly (*p* < 0.001) across all domains, and clinical response was achieved in 40% of patients. Significant improvements were observed in SWN-S and WHO-5 scores (both *p* < 0.001). Weight and BMI significantly decreased (–2.64 kg, *p* = 0.013, and –0.91 kg/m^2^, *p* = 0.006, respectively). Numerical non-significant reductions were found in ASEX (*p* = 0.067) and prolactin levels (–30.7 ng/mL, *p* = 0.077). Overall, treatment was well-tolerated. **Conclusions**: Switching to brexpiprazole was associated with improvements in psychopathological, functional, and physical health domains. These findings support its potential role in real-world, personalized therapeutic strategies for patients with schizophrenia following suboptimal outcomes with prior antipsychotic treatments.

## 1. Introduction

Schizophrenia is a severe, chronic disorder with an estimated lifetime prevalence of 0.5–1% worldwide and a substantial burden in terms of individual suffering, functional disability, and societal costs [1]. According to recent estimates, schizophrenia ranks among the top 15 leading causes of disability [2] and is associated with a reduced life expectancy of 15 to 25 years, mainly due to comorbid medical conditions [3,4] and lifestyle factors, such as poor diet, smoking, and reduced physical activity [5,6].

Although antipsychotic medications are the cornerstone of treatment for schizophrenia, their clinical effectiveness is often limited to the reduction in positive symptoms, such as hallucinations and delusions [7]. Negative symptoms (e.g., anhedonia, apathy, avolition, and social withdrawal) and cognitive dysfunctions tend to be more treatment-resistant and represent critical barriers to functional recovery and satisfactory quality of life [8,9]. Moreover, approximately 20–30% of patients fail to respond adequately to a first-line antipsychotic trial and may require long-term polypharmacotherapy [10,11], which is often complicated by adverse effects with impaired adherence and quality of life [12,13].

In this context, switching antipsychotic treatment represents a common and often necessary strategy, typically driven by suboptimal treatment response, the emergence of adverse effects or adherence issues [10]. Lack of efficacy and poor tolerability emerge as the most frequent reasons for change, albeit with some sex-specific differences [14]. Within the range of switching strategies described in the literature, cross-titration and, particularly, the plateau method (i.e., delayed tapering of the previous antipsychotic until the new one has reached a therapeutic level) have been identified as the preferred approach to mitigate withdrawal, rebound, or dopaminergic destabilization, especially when transitioning from full D2-receptor antagonists to partial agonists [15].

Brexpiprazole is a dopamine–serotonin partial agonist [16] that combines antipsychotic efficacy with a favorable tolerability profile, including a lower incidence of extrapyramidal symptoms, metabolic disturbances, sedation, and hyperprolactinemia [7,17,18]. These characteristics, along with its flexible dosing schedule [19], make it a potential candidate for personalized treatment strategies, particularly in patients with comorbidities or suboptimal response to previous antipsychotic regimens [20,21,22,23]. Although the overall antipsychotic effectiveness is comparable to that of other atypical antipsychotics used for schizophrenia [21], treatment with brexpiprazole has been associated with lower rates of therapeutic discontinuation and fewer symptom exacerbations in clinical practice, with improved adherence and quality of life [24]. Indeed, post hoc analyses and real-world studies suggest that brexpiprazole may exert beneficial effects on domains often under-addressed by conventional antipsychotics, including subjective well-being and life engagement—a multidimensional construct encompassing emotional, cognitive, social, and physical functioning [25,26]. Life engagement has emerged as a relevant outcome reflecting patients’ life-fulfillment, participation in meaningful activities, and social interactions, with potential implications for functional recovery and quality of life [27].

Nevertheless, despite its favorable clinical profile, real-world data on brexpiprazole’s effectiveness and safety after switching from other antipsychotics remain limited and warrant further investigation. Therefore, this real-world, observational study retrospectively investigates the effects of switching from first-/second-generation antipsychotics to brexpiprazole in patients with schizophrenia. The primary aim was to evaluate the effects of a three-month treatment on psychotic symptoms, focusing on *Patient Life Engagement*, intended as a proxy of patients’ active and meaningful involvement in daily life. Changes in sexual functioning, well-being and subjective experience with treatment, as well as the adherence and the overall safety/tolerability profile of brexpiprazole, were also evaluated.

## 2. Materials and Methods

### 2.1. Participants

Patients who had been consecutively referred to the Department of Psychiatry at Fondazione Policlinico Universitario “Agostino Gemelli” IRCCS in Rome, between January 2019 and June 2023, with a primary diagnosis of schizophrenia according to DSM-5 criteria [28] were retrospectively screened for inclusion. Eligible participants were outpatients of both sexes who had been receiving maintenance treatment with a first- or second-generation antipsychotic at an adequate dosage for an appropriate duration, and who had subsequently been switched to flexible doses of brexpiprazole (2–4 mg/day) due to partial response and/or tolerability issues, in accordance with the locally approved Summary of Product Characteristics and relevant clinical guidelines [15,19]. Additional inclusion criteria were age between 18 and 65 years and presence of clinically relevant psychotic symptoms at baseline, as indicated by total scores on the Positive and Negative Syndrome Scale (PANSS) prior to the antipsychotic switch [29].

All patients were converted from previous antipsychotic(s) to brexpiprazole monotherapy through a gradual cross-titration over four weeks [15,30]. Specifically, brexpiprazole was administered orally once daily starting at 1 mg/day, increased to 2 mg/day after seven days and, eventually, it was further increased up to the maximum dosage according to clinicians’ judgment, while the previous antipsychotic was gradually decreased using the 25% x week rule and discontinued within four weeks. Concomitantly, continuous psychosocial support was provided. The concurrent use of other psychotropic medications (e.g., anticonvulsants/mood stabilizers, antidepressants, anxiolytics/sedative-hypnotics) was permitted as long as these were not modified throughout the observation period. Exclusion criteria were: the use of any other antipsychotic agents during the study period; neurological disorders associated with cognitive impairment (e.g., intellectual disability, and all forms of dementia, including Alzheimer’s disease, frontotemporal dementia, dementia with Lewy bodies, vascular dementia, Parkinson’s disease-related dementia, and post-traumatic brain injury); current abuse of alcohol or other psychotropic substances (except for tobacco use). By contrast, the lifetime presence of a substance use disorder was not considered an exclusion criterion.

Anonymity was guaranteed to all participants who provided a written informed consent before inclusion. The study was conducted in accordance with the Declaration of Helsinki and was approved by the Institutional Review Board’s ‘Comitato Etico Territoriale (CET) Lazio area 3’ of Rome, Italy, on 14 December 2023 with protocol code 6222.

### 2.2. Procedures and Assessment

Patients’ clinical records were consulted to retrieve sociodemographic (age, gender, educational level, occupation, marital status) and clinical information (body weight, Body Mass Index [BMI], prolactin levels, fasting glucose and lipid profile, smoking habits, history of family psychiatric illness, medical and psychiatric comorbidities, age at onset of schizophrenia, psychiatric hospitalizations, previous antipsychotic drug and class, other current psychopharmacological treatment). Clinical and psychometric data were extracted from charts according to visits regularly performed during the routine clinical practice, i.e., at first assessment (baseline) and at different timepoints, after four and twelve weeks (endpoint).

The primary outcome was the evaluation of psychotic symptoms using the Patient Life Engagement (PLE) score. Unlike the traditional PANSS subscales (i.e., positive, negative, and general psychopathology) that primarily focus on symptom dimensions, the PLE score is a composite measure derived from selected items of the PANSS that provides a multidimensional assessment of patients’ engagement with daily life. It encompasses four core domains and offers a more integrated perspective on how psychotic symptoms affect everyday functioning and initiative. Specifically, it comprises 14 PANSS items grouped into cognitive (P2, conceptual disorganization; N5, difficulty in abstract thinking; N7, stereotyped thinking; G11, poor attention; G15, preoccupation), emotional (N1, blunted affect; N2, emotional withdrawal; G6, depression), social (N3, poor rapport; N4, passive/apathetic social withdrawal; N6, lack of spontaneity and flow of conversation; G16, active social avoidance) and physical (G7, motor retardation; G13, disturbance of volition) domains.

These items were selected based on previous factor-analytic and clinical work aimed at isolating those symptoms most indicative of a patient’s capacity to engage meaningfully with their internal and external environment [25,31]. For all PLE domains and total scores, higher values indicate greater symptom severity and poorer life engagement. In our sample, internal consistency of the 14 PANSS items composing the PLE was evaluated at baseline and good reliability was detected (Cronbach’s α = 0.848; McDonald’s ω = 0.868). Skewness values for individual items ranged between –0.76 and +0.63 (SE = 0.34), confirming approximate normality and excluding floor or ceiling effects.

At the endpoint, patients were classified as responders if they achieved a ≥40% reduction in the total PANSS score from baseline, a threshold representing a clinically meaningful improvement beyond minimal (≥20%) or moderate (≥30%) change, as previously validated [32,33]. Remission was defined as a score of ≤3 (mild severity or less) on each of the following items: P1 (delusions), P2 (conceptual disorganization), P3 (hallucinatory behavior), N1 (blunted affect), N4 (passive/apathetic social withdrawal), N6 (lack of spontaneity and flow of conversation), G5 (mannerisms and posturing), and G9 (unusual thought content) [34,35]. Given that the conventional definition requires symptom stability for ≥6 months, and that assessments in this study were conducted over 12 weeks, remission was accordingly classified as “early remission”. PANSS and PLE ratings were performed by psychiatrists with at least five years of clinical experience and inter-rater consistency was ensured through weekly consensus meetings.

Secondary outcome measures were investigated at the same time-points through the Italian versions of the following self-report questionnaires: the Arizona Sexual Experience Scale (ASEX), a five-item instrument assessing sexual functioning across domains such as drive, arousal, and satisfaction with a total score cut-off ≥ 19 conventionally used for significant dysfunction [36,37]; the Subjective Well-being under Neuroleptic Scale (SWN-S), which evaluates patients’ perceived well-being during antipsychotic treatment, including emotional regulation, self-control, and mental functioning [38]; and the 5-item World Health Organization Well-Being Index (WHO-5), a brief measure of overall psychological well-being and quality of life [39], with total scores of ≤13 and >13 indicating, respectively, poor and good well-being, and a ≥10-point increase from baseline representing a clinically meaningful improvement [40].

Safety and tolerability of brexpiprazole were confirmed by physical examination, measurement of body weight and vital signs, routine laboratory and instrumental clinical tests, and patients’ reports of any adverse events, as recorded in clinical charts.

### 2.3. Statistical Analysis

Descriptive data were summarized as the number of patients and percentage (%) or mean and standard deviation (M ± SD). The outcome measures—defined as mean changes from baseline to 1 and 3 months for each efficacy variable—were analyzed using repeated measures analysis of variance (ANOVA), with time as the within-subjects factor. Age, gender, and psychiatric comorbidity were included as between-subjects covariates to adjust for potential confounding effects. Assumptions of repeated-measures ANOVA were checked: normality of residuals was verified graphically, and sphericity was tested with Mauchly’s test; when the assumption of sphericity was violated, Greenhouse–Geisser corrections were applied. When the main effect of time was statistically significant, pairwise comparisons between time points were conducted with Bonferroni correction for multiple testing. Effect sizes were reported using partial eta squared (η^2^). Beyond Bonferroni correction for within-outcome pairwise comparisons, multiplicity across primary and secondary endpoints was controlled using the Benjamini–Hochberg false discovery rate (FDR) procedure (q = 0.05). For each outcome, standardized effect sizes (Cohen’s d) with 95% confidence intervals were calculated for baseline-to-endpoint changes to quantify the magnitude of improvement.

Sensitivity to clinical change in the primary outcome measure (i.e., PLE) was assessed by computing paired-sample Cohen’s d for baseline-to-endpoint differences, showing large effect sizes for both total (d = 3.61) and domain scores (cognitive d = 3.48; emotional d = 2.78; social d = 2.72; physical d = 1.97). Convergent validity between PLE and scales capturing subjective well-being and quality of life, conceptually related to life engagement (i.e., SWN-S and WHO-5), was tested through Pearson’s correlations, which revealed significant baseline associations between PLE and SWN-S (r = –0.37, *p* = 0.009), but not with WHO-5 (r = –0.15, *p* = 0.31).

For clinical parameters (i.e., body weight, BMI, prolactin levels, fasting glucose, total cholesterol, high-density [HDL], and low-density [LDL] lipoproteins), changes from baseline to endpoint were tested using paired samples t-tests or Wilcoxon Signed Ranks Tests, according to data distribution. Post hoc exploratory analyses were performed to evaluate the potential impact of prior antipsychotic class (stratified as prolactin-elevating vs. non-prolactin-elevating profile and high vs. low/neutral metabolic liability) and concomitant psychotropic medications on specific outcomes (i.e., PLE/PANSS, ASEX, weight/BMI and prolactin levels). Between-group comparisons were conducted using repeated-measures ANOVAs with ‘Time × Group’ interactions (for PLE, PANSS and ASEX) and independent-samples t-tests (for Δ weight, BMI, prolactin).

Analyses were performed on all patients with at least one valid post-baseline assessment of the variables (full-analysis set, FAS). A significance level of 0.05 was used for each test. All analyses were conducted using IBM SPSS Statistics for Windows, v. 28.0.

## 3. Results

One hundred and sixteen patients referred for schizophrenia were screened for enrollment and, after removing those who did not fulfill the inclusion criteria (n = 32), presented missing data in their medical charts (n = 20), or refused to participate (n = 14), a total of 50 Caucasian subjects who switched to brexpiprazole from previous antipsychotic treatments were included. Of them, 17 (34%) were switched due to partial response, 24 (48%) due to adverse effects, and 9 (18%) due to both reasons. The mean brexpiprazole dose was 3.04 ± 0.71 mg/day. Demographic, clinical, and psychometric characteristics at baseline are summarized in Table 1.

The following psychiatric comorbidities were identified in 36.7% of the sample: major depressive episodes (63.3%), anxiety disorders (18.2%), eating disorders (9.1%), and adult attention-deficit/hyperactivity disorder (9.1%). In addition, substance use disorders in current remission were detected in 46.4% of participants, specifically: polysubstance use 46.2%, cocaine 23.1%, alcohol 15.4%, and cannabinoids 15.3%.

At baseline, PANSS scores were 120 ± 11.2 for the total, 25.1 ± 4.75, 29.7 ± 4.07 and 65.7 ± 6.76, respectively, for ‘positive’, ‘negative’ and ‘general psychopathology’ subscales. At the endpoint, data were available for 40 subjects with all dropouts occurring after the first month (drop-out rate: 20%, n = 10, 95% CI: 11.2–33.0). According to available clinical records, none of these cases reflected pharmacological discontinuation due to inefficacy or adverse effects and were attributed to non-clinical reasons.

Changes in psychotic symptoms expressed as mean PLE total and domains scores are reported in Table 2.

Given the statistically significant ‘Time × Gender’ interaction observed for the cognitive PLE domain (*p* = 0.029), a supplementary analysis examined gender differences in symptom trajectories across timepoints. Estimated marginal means revealed that female patients reported greater impairment in cognitive life engagement at baseline (21.7, 95% CI: 19.99–23.4) compared to males (18.9, 95% CI: 17.58–20.3). Both groups showed substantial improvements at a one-month follow-up (females: 16.7, 95% CI: 14.87–18.6 vs. males: 15.8, 95% CI: 14.34–17.3), with final scores converging at the endpoint (11.1, 95% CI: 9.42–12.7 in females; 11.3, 95% CI: 10.04–12.6 in males).

**Figure 1:**
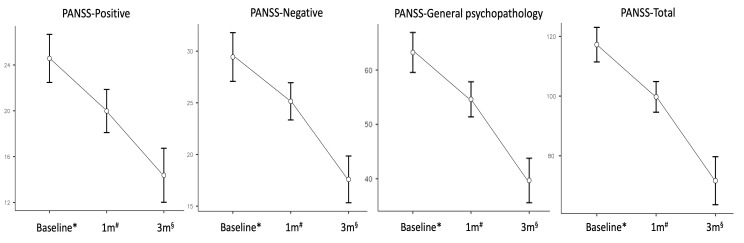
Changes in mean Positive and Negative Syndrome Scale scores with standard errors at different timepoints (ANOVA). Abbreviations: 1 m, one month follow-up; 3 m, three months follow-up; ANOVA, Repeated Measures Analysis of Variance; PANSS, Positive and Negative Syndrome Scale; sample size at * baseline n = 50, # one month n = 50, § endpoint n = 40.

Changes in psychotic symptoms expressed as mean PANSS scores, evaluated after one and three months of treatment, are reported in Figure 1. Reductions at each timepoint were significant after Bonferroni correction (*p* < 0.001) and were the following for the ‘positive’, ‘negative’, ‘general psychopathology’ and total scores, respectively: 20.0 ± 0.89, 25.1 ± 0.85, 54.6 ± 1.53, 99.7 ± 2.44 after one month; 14.4 ± 1.12, 17.6 ± 1.07, 39.7 ± 1.94, 71.6 ± 3.81 at three months. None of the covariates displayed a significant interaction with symptomatic changes over time. At the endpoint, 40% of patients met response criteria defined as a ≥40% reduction in PANSS total score (95% CI: 27.6–53.8), 22% (95% CI: 12.8–35.2) achieved early remission, while the proportion of non-responders was 18% (95% CI: 9.8–30.8).

Exploratory analyses according to prior antipsychotic class observed a significant ‘Time × Group’ interaction for the PANSS total score (*p* = 0.036), indicating greater improvement among patients switched from prolactin-elevating compounds (endpoint marginal means: 63.3, 95% CI: 55.2–71.5) compared to those from non-elevating drugs (77.3, 95% CI: 72.5–82.0). A similar pattern emerged for the PLE total score (*p* = 0.008), with greater reduction (=improvement) in the prolactin-elevating group (30.2, 95% CI: 23.2–37.2) vs. the non-elevating group (34.4, 95% CI: 31.4–37.5). A modest effect was also detected by metabolic liability (*p* = 0.046), with slightly lower PLE total scores (=better) in patients switched from high-risk agents (32.9, 95% CI: 27.9–38.0) compared to low-risk agents (34.2, 95% CI: 30.8–37.6). No significant ‘Time × Group’ interactions were found for concomitant medications. However, endpoint scores were slightly lower (=better) among mood stabilizer users for PLE (32.0 vs. 35.9; *p* = 0.006) and among antidepressant users for PANSS (70.3 vs. 79.6; *p* = 0.044).

Improvements from baseline to endpoint were also observed in all secondary outcome measures (i.e., ASEX, SWN-S, and WHO-5 scales) and are reported in Table 3. At baseline, 74% of patients were above the ASEX threshold suggestive of sexual dysfunction, which lowered to 58% at one month and to 32.3% at three months. According to the WHO-5 cut-off, the proportion of patients above the ‘good well-being’ threshold was only 4% at baseline, which increased to 25% at one month and to 96.8% at three months, while 25.8% achieved a ≥10-point improvement from baseline to endpoint.

Given the significant main effect of ‘Gender’ on ASEX scores, a supplementary analysis to estimate sex-stratified marginal means showed that female patients reported higher ASEX scores at baseline (23.1, 95% CI: 20.3–25.9) compared to males (19.6, 95% CI: 17.4–21.8). This pattern persisted at one month (females: 21.0, 95% CI: 18.7–23.4 vs. males: 18.2, 95% CI: 16.4–20.1) and three months (females: 19.6, 95% CI: 17.2–21.9 vs. males: 16.7, 95% CI: 14.8–18.5), despite general improvement over time in both groups.

Mean values (SD) at baseline, after one and three months of treatment for both primary and secondary outcome measures are reported in Supplementary Table S1. All improvements remained significant after controlling for multiple comparisons using the Benjamini–Hochberg false discovery rate (q ≤ 0.012 across 12 endpoints). Effect sizes were consistently large for both primary (PLE and PANSS) and secondary (ASEX, SWN-S, WHO-5) outcomes, supporting the robustness and clinical relevance of the observed changes (see Supplementary Table S2 for d values and 95% CIs).

Mean changes (SE) from baseline to endpoint observed in laboratory parameters were −2.64 (0.91) for weight [t = −2.9; *p* = 0.013], −0.91 (0.27) for BMI [t = −3.37; *p* = 0.006], and −30.7 (11.6) for prolactin levels [t = −2.65; *p* = 0.077]. Further exploratory analyses showed no significant differences in weight, BMI, or prolactin changes based on prior treatment class or concomitant medications (all *p* > 0.10). No significant changes were observed at endpoint in fasting glucose [−2.57 (3.65), t = −0.71, *p* = 0.508], total cholesterol [−1.5 (6.48), t = −0.23, *p* = 0.826], HDL [0.5 (3.69), t = 0.13, *p* = 0.898], and LDL [−0.67 (2.58), t = −0.26, *p* = 0.806], suggesting a metabolically neutral profile over the 12-week period.

A minority of patients (n = 5, 10%) reported mild side effects (i.e., akathisia, dizziness/drowsiness) within the first month of treatment that gradually disappeared over time and did not lead to treatment discontinuation in any case.

## 4. Discussion

In this real-world, observational study, the switch to brexpiprazole in patients with schizophrenia who had previously been treated with first- or second-generation antipsychotics was associated with improvements in both clinical and functional outcomes over the 12-week follow-up. Notably, Patient Life Engagement (PLE) significantly improved across cognitive, emotional, social, and physical domains, reflecting a broader recovery in dimensions crucial to daily functioning and autonomy. These changes were paralleled by enhanced subjective well-being and quality of life, with a trend toward improvement in sexual functioning and an overall favorable safety profile.

Life engagement refers to the degree to which individuals are actively involved in meaningful activities and social roles that support their sense of identity, purpose, and well-being [25,26]. In schizophrenia, these domains are frequently impaired due to the interplay of negative symptoms, cognitive deficits, and social dysfunctions—factors that are closely linked to reduced functional capacity, lower quality of life, and poor long-term outcomes, even when positive symptoms remit [3,8,9]. Although symptom-focused scales, such as the PANSS, remain fundamental in the clinical assessment of schizophrenia, they may not adequately capture domains related to recovery and reintegration [32,33,35]. Conversely, constructs such as PLE provide a complementary framework to traditional psychopathological evaluations by encompassing subjective well-being and functional restoration. This perspective aligns with modern paradigms of patient-centered and recovery-oriented care [41,42,43]. Recent studies have employed PANSS-derived proxies to operationalize PLE in schizophrenia, supporting the preliminary validity of this approach and emphasizing the increasing focus on outcomes that are personally meaningful to patients [25].

The effects of brexpiprazole on PLE, particularly in the cognitive and social domains, may reflect its distinct pharmacodynamic profile [21,24]. Unlike other compounds with a predominant dopaminergic activity, brexpiprazole exerts a partial agonism at dopamine D2 and serotonin 5-HT1A receptors, and an antagonism at serotonin 5-HT2A and noradrenergic receptors. Its mechanism of action may support executive functioning and attention regulation, as well as mood stabilization and motivational drive, potentially reflecting a reduction in negative symptom burden—typically less responsive to standard antipsychotic treatment [17,44]. Exploratory analyses suggested a greater magnitude of cognitive improvement among female patients who appeared to benefit more markedly in life-engagement–related cognitive functioning, although endpoint values were comparable with males. While this effect should be interpreted cautiously, given the post hoc nature of the analysis and warrants further investigation, it may reflect gender-related differences in baseline functioning or treatment responsiveness, as reported in prior literature [45].

The pharmacodynamic profile of brexpiprazole can provide a plausible explanation for the observed improvements in life engagement, but alternative interpretations cannot be excluded. Cognitive gains may partly reflect regression to the mean or relief from residual sedation and anticholinergic burden associated with prior treatments. Social improvements could also derive from the alleviation of adverse effects that interfere with interpersonal functioning (e.g., extrapyramidal symptoms, sexual side effects), or from non-specific factors such as enhanced clinical monitoring and support during treatment transition. Accordingly, favorable changes in patient-reported outcomes were detected with improvements in subjective well-being across both the SWN-S and WHO-5 scales, which are increasingly used to assess emotional and functional recovery [38].

Notably, the present study is among the few naturalistic investigations to employ a validated self-rated scale to assess sexual functioning in patients with schizophrenia [36]. Although the overall reduction in ASEX scores did not reach statistical significance, the data show a potential trend toward improved sexual functioning. Additionally, female patients reported persistently higher ASEX scores, suggesting a greater burden of sexual dysfunction despite overall improvements. This exploratory observation is consistent with existing literature highlighting gender differences in the subjective experience and reporting of sexual side effects during antipsychotic treatment [46] and underscores the importance of gender-sensitive monitoring in clinical practice. Indeed, depending on the pharmacological profile and individual characteristics, sexual dysfunctions can affect up to 80% of patients treated with antipsychotics and are strongly associated with reduced quality of life and poor treatment adherence [47,48]. Nevertheless, they remain underreported and overlooked in clinical trials that generally lack systematic use of specific assessment tools. Moreover, recent expert consensus highlights that, in the context of functional recovery, improvements in domains such as depression and social interaction are often prioritized over others, including leisure and sexual functioning, by both clinicians and patients [49], thus partially explaining why sexual health is not consistently addressed in routine assessments.

In parallel, a trend-level reduction in serum prolactin concentration was observed. Given the well-established association between hyperprolactinemia and sexual dysfunction—including decreased libido, erectile and orgasmic difficulties, amenorrhea, and bone demineralization—these findings, although preliminary, hold clinical relevance [50]. Prolactin elevation is commonly induced by dopamine D2 receptor antagonism in the tuberoinfundibular pathway, which disrupts tonic inhibition of anterior pituitary secretion [51]. Brexpiprazole’s partial agonism at these receptors, combined with its low intrinsic activity, may preserve dopaminergic tone and, thus, attenuate prolactin elevation [52]. Additionally, its negligible affinity for serotonergic receptors involved in prolactin release likely contributes to a favorable endocrine profile [53]. These pharmacodynamic features offer a plausible mechanistic explanation for the observed trends in both sexual and endocrine parameters, supporting the need for further investigation.

The present findings also support the overall metabolic neutrality of brexpiprazole. Reductions in weight and BMI following the switch are encouraging, considering that metabolic syndrome is a major contributor to excess mortality in schizophrenia [54]. While antipsychotic-induced metabolic disturbances are well documented, recent metabolomic and epidemiological analyses emphasize that such abnormalities in schizophrenia stem from complex interactions between genetic predisposition, environmental exposures, and disease-specific mechanisms [55,56]. Further, there is growing recognition of an intrinsic vulnerability to metabolic dysregulation in patients with schizophrenia, independent of pharmacologic exposure [3,4]. Evidence from drug-naive, first-episode patients reveals elevated prevalence of insulin resistance, dyslipidemia, and visceral adiposity, suggesting shared pathophysiological pathways involving inflammation, hypothalamic–pituitary–adrenal axis disruption, and mitochondrial alterations [22,23,57]. In this context, brexpiprazole’s limited affinity for histaminergic and muscarinic receptors may underlie its lower metabolic liability, in line with prior prospective studies and meta-analyses highlighting the relative metabolic neutrality of D2 partial agonists [13]. Furthermore, in our cohort, improvements in psychotic and engagement-related symptoms appeared to be more pronounced among patients previously treated with prolactin-elevating antipsychotics, whereas changes in metabolic and endocrine parameters were largely independent of prior treatment class. This pattern may suggest that brexpiprazole’s partial agonism at dopamine D_2_ receptors not only mitigates hyperprolactinemia but may also potentially support global functioning in patients transitioning from potent antagonists.

Approximately one-third of patients in our sample achieved early symptomatic remission, while nearly half met the response threshold based on standard PANSS-derived cut-offs [34]. These results are consistent with those reported in randomized controlled trials and real-world studies evaluating second-generation antipsychotics [11,33,35]. The efficacy profile of brexpiprazole appears broadly comparable to that of other antipsychotic agents examined in large-scale meta-analyses, particularly about symptom control and global functioning [7,18,44]. Nevertheless, our findings are encouraging given the naturalistic design and the inclusion of patients with partial response or poor tolerability to previous treatments. It is plausible that the observed response pattern may also reflect brexpiprazole’s effectiveness in individuals with complex clinical presentations including psychiatric comorbidities, because of its multimodal receptor activity that combines antipsychotic efficacy with mood-stabilizing and anxiolytic properties [20,21]. Indeed, prior switching studies have demonstrated the feasibility and clinical benefit of transitioning to brexpiprazole, supporting its use in flexible and individualized therapeutic strategies [15]. Notably, no differential trajectories were observed according to concomitant use of antidepressants or mood stabilizers, supporting the robustness and generalizability of treatment effects across common co-therapy regimens.

Finally, although adverse events were recorded narratively based on clinical observation and patient reports without the administration of standardized rating scales for extrapyramidal symptoms or akathisia, brexpiprazole exhibited a favorable tolerability profile with minimal reports of sedation, akathisia, or extrapyramidal symptoms. This finding aligns with previous safety data and supports its suitability for long-term use, particularly in patients at risk of poor adherence due to adverse effects [16,48]. Considering the well-established association between tolerability and adherence [10], this aspect is of central importance in real-world effectiveness. Brexpiprazole’s safety profile is further corroborated by its pharmacodynamic characteristics and by comparative analyses indicating superior tolerability among dopamine partial agonists [17,24,58]. Collectively, these findings support brexpiprazole as a viable treatment option for patients requiring improved balance between tolerability and efficacy. Further, although clozapine remains the gold standard for treatment-resistant cases of schizophrenia, augmentation or switching strategies are often required, and growing evidence, albeit limited, highlights the potential role of third-generation antipsychotics, including brexpiprazole, also in similar contexts [59,60,61].

Several limitations warrant consideration in this study. The retrospective and monocentric design, the relatively short follow-up duration of 12 weeks, the small sample size, and the absence of a control group may limit the interpretation of treatment effects. In addition, the clinical heterogeneity of prior antipsychotic exposure may have influenced outcomes and prevented comparisons between pharmacologically similar subgroups. However, exploratory subgroup and sensitivity analyses accounting for prior antipsychotic class and concomitant medications supported the robustness of the main findings, suggesting that improvements in PANSS and PLE were not merely attributable to withdrawal from previous treatments and appeared consistent across common co-therapy regimens. The sample also consisted exclusively of Caucasian patients, further limiting ethnic diversity. Another methodological limitation concerns the use of repeated-measures ANOVA rather than linear mixed-effects models, which represent the optimal analytic approach in the presence of attrition. However, in our dataset, drop-outs did not differ from completers at baseline, and sensitivity analyses (per-protocol and last-observation-carried-forward) confirmed the robustness of the primary results, mitigating concerns about attrition bias. Taken together, these aspects may constrain the external validity of the results. Nevertheless, the real-world nature of treatment decisions enhances their relevance, and future research including more diverse populations, longer follow-up periods, and controlled study designs will be essential to confirm and extend the generalizability of our findings.

Despite these aspects, the study offers several strengths. It is among the first to adopt a proxy measure of Patient Life Engagement, derived from PANSS items, alongside conventional PANSS-based response and remission criteria. The inclusion of sexual functioning evaluation through a validated self-report instrument, seldom used in naturalistic switch studies, further contributes to the originality of the work. The integration of clinical, functional, and patient-reported outcomes provides a broad and clinically relevant perspective on brexpiprazole’s effects following antipsychotic switching.

## 5. Conclusions

Schizophrenia is a chronic and disabling psychiatric disorder often marked by persistent negative symptoms, cognitive deficits and social dysfunction, which substantially impact long-term functioning and quality of life, even in individuals achieving remission of positive symptoms [5,8]. Optimizing the balance between efficacy, tolerability, and functional recovery remains a core therapeutic goal [9].

In this real-world study, switching to brexpiprazole from other antipsychotics was associated with improvements in both psychopathological symptoms and patient-centered outcomes, like patient life engagement and subjective well-being. Metabolic parameters such as weight and BMI also showed favorable changes, alongside a trend toward reduced prolactin levels. The treatment was generally well tolerated, with a low incidence of adverse effects. Although sexual functioning did not significantly improve, a positive trend was noted, reinforcing the importance of addressing this often-neglected aspect of care. In conclusion, these findings support brexpiprazole as a treatment option for individuals requiring optimization of antipsychotic regimens and prioritizing improved tolerability and attention to patient-centered outcomes. The use of a proxy measure for life engagement, combined with standardized clinical criteria and validated self-report instruments, provides an integrated and complementary perspective on treatment response. Future, prospective research with longer follow-up and broader functional evaluations is needed to confirm and extend these results within personalized care strategies for schizophrenia.

## Figures and Tables

**Table 1:** Sociodemographic and clinical characteristics at baseline.

Characteristics (n, %; M ± SD)	
**Overall**	50
*Sociodemographic*	
**Age** (years)	41.5 ± 15.1
**Gender**	
Male	30 (60)
Female	20 (40)
**Educational level** (years)	15 ± 3.06
**Occupation**	
Employed	34 (68.7)
Unemployed	16 (31.3)
**Marital status**	
Married	12 (23.5)
Unmarried	38 (76.5)
*Clinical*	
**Smoking habits** (yes)	30 (60)
**Medical comorbidities** (yes)	14 (28)
**Weight** (kg)	81.5 ± 17.6
**BMI** (kg/m^2^)	25.7 ± 4.34
**Prolactin** (ng/mL)	33.2 ± 23.3
**Glucose** (mg/dL)	83.0 ± 7.0
**Cholesterol** (mg/dL)	213.2 ± 68.7
**HDL** (mg/dL)	56.8 ± 17.7
**LDL** (mg/dL)	138.2 ± 57.0
**Family psychiatric history** (yes)	33 (66.7)
**Age at onset** (years)	27.9 ± 10.9
**Psychiatric hospitalizations** (yes)	17 (34.5)
**Previous antipsychotic drug**	
Amisulpride	3 (6)
Aripiprazole	11 (22)
Asenapine	1 (2)
Clozapine	1 (2)
Haloperidol	3 (6)
Lurasidone	4 (8)
Olanzapine	19 (38)
Paliperidone	1 (2)
Quetiapine	3 (6)
Risperidone	4 (8)
**Previous antipsychotic class**	
Prolactin-elevating	11 (22)
Non-prolactin-elevating	39 (78)
High metabolic liability	23 (46)
Low/Neutral metabolic liability	27 (54)
**Other psychopharmacotherapy** (yes)	40 (80)
Antidepressants	26 (52.4)
Anticonvulsants/Mood stabilizers	27 (53.5)
Sedative-hypnotics/Anxiolytics	27 (54.8)
*Psychometric*	
**PLE**	58.5 ± 7.26
Cognitive	20.5 ± 2.43
Emotional	13.6 ± 1.98
Physical	7.96 ± 1.52
Social	16.5 ± 2.92
**ASEX**	20.8 ± 3.74
**SWN-S**	77.1 ± 6.59
**WHO-5**	9.54 ± 2.47

Abbreviations: ASEX, Arizona Sexual Experience Scale; BMI, Body Mass Index; M, mean; PLE, Patient Life Engagement score; SD, Standard Deviation; SWN-S, Subjective Well-being under Neuroleptic Scale; WHO-5, World Health Organization-Five Well-Being Index.

**Table 2:** Changes in *Patient Life Engagement* scores at different time-points by age, gender and psychiatric comorbidity as covariates (ANOVA).

PLE			
Cognitive	F	*p*	η^2^p
*Within-Subjects Effects*			
**Time**	10.82	**<0.001**	0.07
Time * Age	0.29	0.752	0.002
Time * Gender	3.95	**0.029**	0.026
Time * Psychiatric Comorbidity	0.41	0.672	0.003
*Between-Subjects Effects*			
**Age**	4.88	**0.042**	0.072
**Gender**	0.69	0.419	0.010
**Psychiatric Comorbidity**	0.53	0.478	0.008
**Emotional**	F	*p*	η^2^p
*Within-Subjects Effects*			
**Time**	7.49	**0.008**	0.028
Time * Age	0.82	0.451	0.004
Time * Gender	0.53	0.592	0.003
Time * Psychiatric Comorbidity	0.46	0.633	0.002
*Between-Subjects Effects*			
**Age**	0.12	0.731	0.002
**Gender**	1.49	0.239	0.027
**Psychiatric Comorbidity**	0.01	0.927	0.000
**Physical**	F	*p*	η^2^p
*Within-Subjects Effects*			
**Time**	3.47	**0.043**	0.039
Time * Age	0.49	0.616	0.005
Time * Gender	0.06	0.943	0.001
Time * Psychiatric Comorbidity	0.16	0.856	0.002
*Between-Subjects Effects*			
**Age**	0.93	0.349	0.014
**Gender**	0.01	0.910	0.003
**Psychiatric Comorbidity**	1.31	0.270	0.020
**Social**	F	*p*	η^2^p
*Within-Subjects Effects*			
**Time**	5.84	**0.007**	0.042
Time * Age	0.25	0.779	0.002
Time * Gender	1.06	0.359	0.008
Time * Psychiatric Comorbidity	0.63	0.538	0.005
*Between-Subjects Effects*			
**Age**	0.13	0.722	0.002
**Gender**	0.34	0.567	0.006
**Psychiatric Comorbidity**	0.41	0.534	0.007
**Total**	F	*p*	η^2^p
*Within-Subjects Effects*			
**Time**	9.45	**<0.001**	0.06
Time * Age	0.53	0.594	0.003
Time * Gender	1.41	0.258	0.009
Time * Psychiatric Comorbidity	0.09	0.912	0.001
*Between-Subjects Effects*			
**Age**	0.04	0.839	0.001
**Gender**	0.11	0.742	0.002
**Psychiatric Comorbidity**	1.18	0.294	0.020

Significant results in **bold**. Abbreviations: η^2^p, partial eta-squared; ANOVA, Repeated Measures Analysis of Variance; F, between- and within-group ratio; *p*, statistical significance; PLE, Patient Life Engagement.

**Table 3:** Changes in sexual functioning, subjective well-being, and quality of life at different time-points by age, gender and psychiatric comorbidity as covariates (ANOVA).

ASEX	F	*p*	η^2^p
*Within-Subjects Effects*			
**Time**	2.95	0.067	0.006
Time * Age	0.29	0.766	0.001
Time * Gender	0.381	0.686	0.001
Time * Psychiatric Comorbidity	0.351	0.707	0.002
*Between-Subjects Effects*			
**Age**	2.55	0.130	0.043
**Gender**	6.19	**0.024**	0.105
**Psychiatric Comorbidity**	0.01	0.947	0.000
**SWN-S**	F	*p*	η^2^p
*Within-Subjects Effects*			
**Time**	11.43	**<0.001**	0.108
Time * Age	0.05	0.949	0.000
Time * Gender	0.47	0.629	0.004
Time * Psychiatric Comorbidity	1.58	0.222	0.015
*Between-Subjects Effects*			
**Age**	0.51	0.485	0.008
**Gender**	0.02	0.896	0.000
**Psychiatric Comorbidity**	0.72	0.412	0.011
**WHO-5**	F	*p*	η^2^p
*Within-Subjects Effects*			
**Time**	24.32	**<0.001**	0.079
Time * Age	0.324	0.725	0.001
Time * Gender	0.253	0.778	0.001
Time * Psychiatric Comorbidity	1.202	0.314	0.004
*Between-Subjects Effects*			
**Age**	0.01	0.695	0.003
**Gender**	0.05	0.831	0.001
**Psychiatric Comorbidity**	1.85	0.191	0.024

Significant results in **bold**. Abbreviations: η^2^p, partial eta-squared; ANOVA, Repeated Measures Analysis of Variance; ASEX, Arizona Sexual Experience Scale; F, between- and within-group ratio; *p*, statistical significance; SWN-S, Subjective Well-being under Neuroleptic Scale; WHO-5, World Health Organization-Five Well-Being Index.

## Data Availability

The authors do not have permission to share these data.

## Supplementary Materials

The following supporting information can be downloaded at: https://www.mdpi.com/article/10.3390/jpm15110502/s1, Table S1: Values of primary and secondary outcome measures at different time-points; Table S2: Baseline-endpoint changes and standardized effect sizes for all outcomes.
