# Supplementary material for: Patient Life Engagement and Metabolic Profile Improve After Switching from First-/Second-Generation Antipsychotics to Brexpiprazole: A Real-World Study in Patients with Schizophrenia
Source: J Pers Med. 2025 Oct 22;15(11):502. doi: 10.3390/jpm15110502 (PMC12653920; doi:10.3390/jpm15110502)
Supplement: Supplementary file 1 [file jpm-15-00502-s001.zip › Supplementary Table S1.pdf]

**Supplementary Table S1.** Values of primary and secondary outcome measures at different time-points.

| *Mean values ± SD* | Baseline (n = 50) | One month (n = 50) | Three months (n = 40) |
|---|---|---|---|
| **PANSS** | | | |
| Positive | 25.1 ± 4.75 | 20.4 ± 4.2 | 14.5 ± 4.2 |
| Negative | 29.7 ± 4.07 | 25 ± 3.6 | 18.2 ± 3.9 |
| General psychopathology | 65.7 ± 6.76 | 55.9 ± 6.8 | 42.1 ± 8.1 |
| Total | 120 ± 11.2 | 101 ± 11 | 74.8 ± 15.1 |
| **PLE** | | | |
| Cognitive | 20.5 ± 2.43 | 16.4 ± 2.2 | 11.6 ± 2 |
| Emotional | 13.6 ± 1.98 | 11.6 ± 1.7 | 8.5 ± 1.6 |
| Physical | 7.96 ± 1.52 | 6.6 ± 1.2 | 4.6 ± 0.9 |
| Social | 16.5 ± 2.92 | 13.2 ± 2.5 | 8.9 ± 1.6 |
| Total | 58.5 ± 7.26 | 47.9 ± 6.5 | 33.6 ± 5.1 |
| **ASEX** | 20.8 ± 3.74 | 19.2 ± 3.16 | 17.7 ± 3.03 |
| **SWN-S** | 77.1 ± 6.59 | 85.9 ± 7.4 | 98.7 ± 4.9 |
| **WHO-5** | 9.54 ± 2.47 | 13.4 ± 2.16 | 17.8 ± 2.33 |

Abbreviations: ASEX, Arizona Sexual Experience Scale; PANSS, Positive and Negative Syndrome Scale; PLE, Patient Life Engagement; SD, Standard Deviation; SWN-S, Subjective Well-being under Neuroleptic Scale; WHO-5, World Health Organization-Five Well-Being Index.
