# Supplementary material for: Patient Life Engagement and Metabolic Profile Improve After Switching from First-/Second-Generation Antipsychotics to Brexpiprazole: A Real-World Study in Patients with Schizophrenia
Source: J Pers Med. 2025 Oct 22;15(11):502. doi: 10.3390/jpm15110502 (PMC12653920; doi:10.3390/jpm15110502)
Supplement: Supplementary file 1 [file jpm-15-00502-s001.zip › Supplementary Table S2.pdf]

Supplementary Table S2. Baseline-endpoint changes and standardized effect sizes for all outcomes.

Outcome	Cohen's *d* (95% CI)	p	q
**PANSS**			
Positive	2.41 (1.72–3.08)	<0.001	≤0.012
Negative	2.50 (1.80–3.20)	<0.001	≤0.012
General psychopathology	2.67 (1.93–3.40)	<0.001	≤0.012
Total	2.95 (2.15–3.74)	<0.001	≤0.012
**PLE**			
Cognitive	3.48 (2.55–4.41)	<0.001	≤0.012
Emotional	2.78 (2.00–3.54)	<0.001	≤0.012
Physical	1.97 (1.37–2.57)	<0.001	≤0.012
Social	2.72 (1.96–3.48)	<0.001	≤0.012
Total	3.61 (2.65–4.57)	<0.001	≤0.012
**ASEX**	1.35 (0.86–1.84)	<0.001	≤0.012
**SWN-S**	−3.25 (−4.17 to −2.33)	<0.001	≤0.012
**WHO-5**	−3.70 (−4.70 to −2.70)	<0.001	≤0.012

Abbreviations: ASEX, Arizona Sexual Experience Scale; CI, Confidence Intervals; *d*, standardized Cohen's effect sizes; PANSS, Positive and Negative Syndrome Scale; *p*, statistical significance; PLE, Patient Life Engagement; SWN-S, Subjective Well-being under Neuroleptic Scale; WHO-5, World Health Organization-Five Well-Being Index. Signs reflect scale direction (e.g., reductions on PLE/PANSS and ASEX vs increases on WHO-5/SWN); interpretation is based on magnitude and expected clinical direction. Bonferroni correction was retained for within-outcome pairwise time contrasts, as requested. Benjamini–Hochberg FDR (q) was applied across endpoints (m = 12).
